# Feasibility of oral microbiome profiles associated with oral squamous cell carcinoma

**DOI:** 10.1080/20002297.2022.2105574

**Published:** 2022-08-07

**Authors:** Kengo Hashimoto, Dai Shimizu, Sei Ueda, Satoru Miyabe, Ichiro Oh-Iwa, Toru Nagao, Kazuo Shimozato, Shuji Nomoto

**Affiliations:** a Department of Oral and Maxillofacial Surgery, Aichi-Gakuin University School of Dentistry, Nagoya, Japan; b Department of Surgery, Aichi-Gakuin University School of Dentistry, Nagoya, Japan; c Department of Maxillofacial Surgery, Japanese Red Cross, Nagoya Daiichi Hospital, Nagoya, Japan

**Keywords:** Oral squamous cell carcinoma, oral leukoplakia, oral microbiome, saliva, next-generation sequencer

## Abstract

**Objective:**

Recently, the possibility that oral microbiomes is associated with oral squamous cell carcinoma (OSCC) initiation and progression has attracted attention; however, this association is still unclear. Here, we comprehensively analyze the microbiome profiles of saliva samples using next-generation sequencing followed by determining the association between oral microbiome profiles and OSCC.

**Materials and Methods:**

Microbiome profiles in saliva samples from patients with OSCC, oral leukoplakia (OLK), and postoperative OSCC (Post) were analyzed. Candidate OSCC-associated bacteria were identified by comparing the bacterial diversity and relative abundance of each group based on these microbiome profiles, and their applicability as OSCC detection tools were evaluated.

**Results:**

There were significant differences in genus abundances (*Streptococcus, Aggregatibacter*, and *Alloprevotella*) among the groups from saliva samples. In the OSCC group, compared with the OLK and Post groups, abundances of the genus *Fusobacterium*, phylum *Fusobacteria* and phylum *Bacteroidetes* were markedly increased and that of the genus *Streptococcus* and phylum *Firmicutes* were decreased.

**Conclusion:**

The results suggested a strong association of these bacteria with OSCC. Especially, phylum *Fusobacterium* was significantly associated with early recurrence of OSCC. Thus, oral microbiome analysis may have a potential of novel OSCC detection and prognostic tool.

## Introduction

Oral cancer is one of the most common cancers worldwide, and >90% of them are squamous cell carcinoma originating from the oral mucosa. Regardless of the advancements in medical technologies, overall five-year survival rate of patients with oral squamous cell carcinoma (OSCC) is approximately 50% over the last few decades [1]. The majority of OSCCs are macroscopically detectable; however, many of patients with OSCC were discovered when they have already progressed to advanced stage [1]. Diagnosis at an early stage of OSCC has a great impact on the improvement of QOL and prognosis. The development of novel highly reliable detection tool is urgently needed.

Currently, serum markers for OSCC are only supplementarily used and do not always reflect the tumor clinical condition. Therefore, they are not suitable for OSCC screening. Regarding the easiness of sample collection, saliva is considered to be more suitable for screening compared with blood. We focused on the possibility of the clinical application of OSCC-associated oral microbiome analysis. Notably, *Helicobacter pylori* has now been recognized as a carcinogenic agent in gastric cancer and low-grade B-cell MALT gastric lymphoma initiation [2]. Recently, the gut microbiome is associated with various diseases, which can be detected using next-generation sequencing (NGS) [3]. Moreover, characteristic oral microbiome has been reported in patients with gastric and pancreatic cancers [4,5]. However, the diagnostic significance of microbiome associated with a particular disease is still unclear. If oral microbiome analysis can be clinically applied to detect OSCC, new cancer control strategies can be developed, including the prevention of OSCC by probiotics.

Here, we investigated oral microbiome profiles in saliva samples, and examined the feasibility of oral microbiome profiles as novel detection tool for OSCC.

## Materials and methods

### Ethics statement

This study protocol was approved by the institutional review board of the Aichi-Gakuin University School of Dentistry and the Japanese Red Cross Nagoya Daiichi Hospital. The study was performed in accordance with the Helsinki Declaration and Good Clinical Practice. All participants approved and signed written informed consents.

### Sample collection

This study included patients with OSCC (n = 41), oral leukoplakia (OLK; n = 25), and postoperative OSCC (Post; n = 20) who visited the department of Oral and Maxillofacial Surgery, Aichi-Gakuin University Dental Hospital and Japanese Red Cross Nagoya Daiichi Hospital, during March 2016 and October 2018. For analysis, 86 saliva samples were used. Saliva samples were collected using an Oragene® DNA kit (DNA Genotek, Ontario, Canada) immediately after waking up (before tooth brushing and eating breakfast). All samples were stored at −20°C until DNA extraction. Patients who were undergoing treatment for infectious diseases or malignant tumors, using oral wash and taking antibiotics within 3 months were excluded. For the OSCC group, patients with primary cancer without any treatment were included. For the OLK group, the presence of epithelial dysplasia was confirmed by biopsy. For the post group, different OSCC group’s patients who underwent surgery alone >3 months prior were included. In post group, one patient had undergone reconstructive surgery. Table 1 shows the clinical features of the participants in the current study.

**Table 1.** Clinical features of OSCC, OLK and postoperative of OSCC patients.

		OSCC (N = 41)	Post* (N = 20)	OLK (N = 25)
	Mean age, years (range)	67.7(28–92)	68.2 (29–85)	64.4 (29–91)
	Sex			
	Male	30	14	16
	Female	11	6	9
	Smoking			
	Current	5	4	2
	Past/never	36	16	23
	Alcohol drinking			
	Heavy	11	5	4
	Moderate or mild/never	30	15	21
	Mean number of tooth (range)	21.5(0–31)	21.9(5–29)	24.1(5–31)
	OSCC/OLK/Post subsite			
	Tongue	15	10	14
	Gingiva	16	8	9
	Buccal mucosa/floor of mouth	10	2	2
	Dysplasia of OLK			
	Absent	-	-	15
	Present	-	-	10
	Pathological stage of OSCC**			
	I/ II	23	17	-
	III/ IV	18	3	-

Abbreviations: OSCC: oral squamous cell carcinoma; OLK: oral leukoplakia; Post: post operative of OSCC.

* includes 1 reconstructive surgery, and no chemotherapy or radiotherapy patients

** UICC TNM classification 7^th^ edition

### DNA extraction and DNA library construction

The extraction of DNA from saliva was carried out according to the Oragene® DNA kit manufacturer’s protocol. All samples were done at the same time. DNA libraries were constructed by two-step tailed polymerase chain reaction (PCR) amplification with unique barcoded primers in the bacterial 16S rRNA V4 region, using the first primer sets (515 F/806 R), and the following second primer sets (F: 5ʹ-AATGATACGGCCGACCACCGAGATCTACAC-Index2-ACACTCTTTCCCTACACGACGC-3ʹ, R: 5ʹ-CAAGCAGAAGACGGCATACGAGAT-Index1-GTGACTGGAGTTCAGACGTGTG-3’). The first PCR reactions run at 94°C for 2 min, followed by 30 cycles of denaturation at 94°C for 30s, annealing at 50°C for 30s, with elongation at 72°C for 30s and final elongation at 72°C for 5 min. The second PCR reactions (using the first PCR product) run at 94°C for 2 min, followed by 10 cycles of denaturation at 94°C for 30s, annealing at 60°C for 30s, with elongation at 72°C for 30s and final elongation at 72°C for 5 min. The composition of each PCR solution is as follows: 2-μl of DNA template (second PCR product), 1.0 μl of 10X PCR buffer, 0.8 μl of dNTPs (2.5 mM), 0.1 μl of 5 U of Taq DNA polymerase (TaKaRa, Kyoto, Japan), 5.1 μl of DDW, 0.5 μl of forward primer (10 μM) and 0.5 μl of reverse primer (10 μl) for a total volume of 10.0 μl.

### Sequence analysis

Sequence analysis was performed using the Illumina MiSeq sequencer (Illumina Inc., San Diego, CA, USA). Quality filtering was performed by the FASTX toolkit. Sequences that passed quality filtering were merged using the paired-end merge script FLASH. The merged sequences were filtered by fragment length, and only 246–260 bases were used for further analysis. Sequences that passed all filtering were checked for chimeric sequence detection using the USEARCH Uchime algorithm. The non-chimeric sequences were clustered into operational taxonomic units (OTU) using Quantitative Insights into Microbial Ecology (QIIME) with a 97% threshold against reference sequences of the Human Oral Microbiome Database (HOMD, 16S rRNA RefSeq version 15.1). The relative abundances of each oral microbiome were constructed, and group significance of abundances among the saliva samples of the three groups (OSCC, OLK, Post) was tested using Kruskal-Wallis.

### Diversity analyses and linear discriminant analysis of effective size (LEfSe)

To analyze the diversity of bacterial flora among the groups, α and β diversity analyses were performed using QIIME scripts. Analysis indices were PD whole tree, Chao1, observed species and Shannon for α diversity, Unweighted and Weighted Unifrac for β diversity. In diversity analysis, 10 healthy subjects (Mean age; 30.2 years, included 3 females) without smoking or drinking habits or any general medical history were used for control. LEfSe analysis were performed to determine the characteristic bacteria for each group. In the results of LEfSe, particularly high linear discriminant analysis (LDA) scores (>4.0) were extracted and used as candidate for OSCC-associated bacteria. Analyses of samples from early OSCC (early) and advanced OSCC (late) were performed to confirm differences in microbiome profile-associated cancer progression.

### Examination of candidate bacteria for OSCC detection tool

Statistical tests were performed among three groups, matched the ages and having tooth numbers as a substitution for periodontal status. Because there were no significant differences of the LEfSe results between the OLK and Post groups (Data not shown), we treated the OLK and post groups as non-OSCC group (OLK and post). The optimal cut-off values of bacterial relative abundance that could distinguish the OSCC and non-OSCC groups were determined using receiver operating characteristic (ROC) curves. These cut-off values were validated using univariate analysis with age, sex and smoking and drinking statuses of each group. The applicability of these bacterial candidates for OSCC detection was examined using multivariate analysis to eliminate confounding factors. In addition, the probability of these candidate bacterium as prognostic factors was examined using LEfSe and Kaplan-Meier method in patients with early recurrence within 1 year postoperatively.

### Statistical analysis

LEfSe was performed using the Galaxy/Hutlab (huttenhower.sph.harvard.edu) algorithms. Univariate and multivariate analyses were performed using Fisher’s exact test and chi-square test, Logistic regression analysis, respectively. Statistical significance was set at p < 0.05. R statistical software (version 3.4.0) was used.

## Results

Sequence data of bacterial DNA from saliva samples corresponded to a total of 11 phyla, 29 class, 51 order, 94 family and 130 genera. The predominant phyla were *Bacteroidetes* (29.5%), *Firmicutes* (28.9%), *Proteobacteria* (23.7%), *Fusobacteria* (10.5%) and *Actinobacteria* (5.1%). The predominant class were *Bacteroidia* (27.7%), *Bacilli* (16.9%), *Gammaproteobacteria* (12.3%), *Fusobacteriia* (10.5%) and *Betaproteobacteria* (9.4%). The predominant order were *Bacteroidetes* (27.7%), *Lactobacillales* (13.9%), *Pasteurellales* (13.6%), *Fusobacteriales* (10.5%) and *Neisseriales* (9.2%). The predominant family were *Prevotellaceae* (23.0%), *Steptococcaceae* (11.8%), *Pasteurellaceae* (11.4%), *Neisseriaceae* (9.2%) and *Fusobacteriaceae* (9.0%). The predominant genera were *Prevotella* (19.3%), *Streptococcus* (11.8%), *Haemophilus* (11.0%), *Fusobacterium* (9.0%) and *Neisseria* (8.9%). Significant differences were observed among the abundance of the genus *Streptococcus, Aggregatibacter* and *Alloprevotella* in saliva samples when compared among the OSCC, OLK and post groups (Table 2).

**Table 2.** Group significance among OSCC, OLK and post.

Taxonomy	FDR P-value	Bonferroni P-value
p_*Firmicutes;* g*_Streptococcus*	0.001	0.001
p_*Proteobacteria;* g*_Aggregatibacter*	0.001	0.0011
p_*Bacteroidetes;* g*_Alloprevotella* (s__sp._HMT_912)	0.011	0.0315
p*_Bacteroidetes;* g*_Alloprevotella* (s__sp._HMT_473)	0.035	
p_*Firmicutes;* g*_Mogibacterium*	0.035	
p_*Actinobacteria;* g*_Rothia*	0.035	
p_*Actinobacteria;* g*_Corynebacterium*	0.035	
p_*Proteobacteria;* g*_Haemophilus*	0.035	
p_*Firmicutes;* g*_Peptostreptococcus*	0.035	
p_*Fusobacteria;* g*_Fusobacterium*	0.049	

Abbreviations: OSCC: oral squamous cell carcinoma; OLK: oral leukoplakia; Post: post operative of OSCC; FDR: false discovery rate; p: phylum; g: genus.

**Figure 1.**
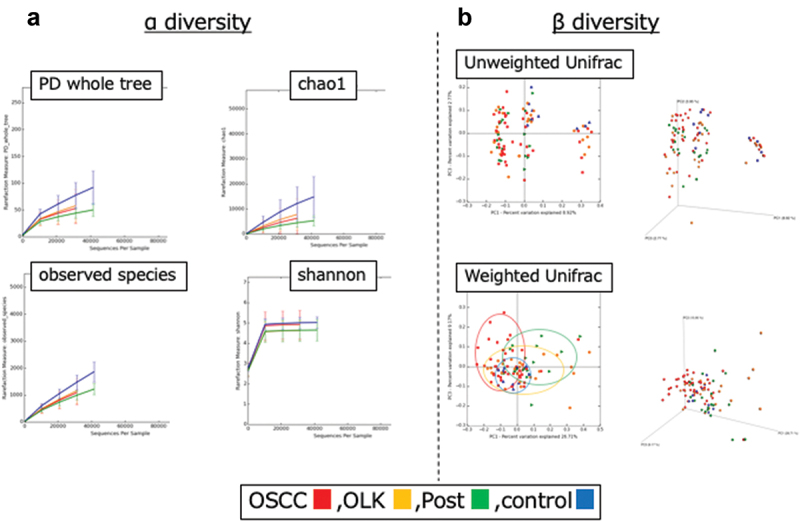
The results of α and β diversity analyses using QIIME scripts for oral squamous cell carcinoma (OSCC), oral leukoplakia (OLK) and post-operative of OSCC (Post) groups. Control group includes 10 healthy subjects without smoking or drinking habits or any general medical history. (a) α Diversity analysis comparing three groups and control. Analysis indices were PD whole tree, Chao1, observed species, and Shannon. (b) β diversity analysis. Analysis indices were Unweighted and Weighted Unifrac.

α diversity analysis revealed that diversity was lowest for the post group; the OSCC and OLK groups had similar diversity (Figure 1(a)). β diversity analysis of weighted unifrac revealed that distribution within a certain range for each group (Figure 1(b)). α diversity analysis of the late and early groups showed greater diversity in the late group (Table 3).

**Table 3.** Summary of α diversity and β diversity of each group.

Procedure of analysis	OSCC, OLK, post, control	OSCC early vs late
Degree of α diversity		
1. PD whole tree	control>OSCC = OLK>Post	late>early
2. chao1	control>OSCC = OLK>Post	late>early
3. observed species	control>OSCC = OLK>Post	late>early
4. shannon	control = OSCC>OLK = Post	late>early
Difference of β diversity		
1. Unweighted Unifrac	-	-
2. Weighted Unifrac	+	+

Abbreviations: OSCC: oral squamous cell carcinoma; OLK: oral leukoplakia; Post: post operative of OSCC; early: stage I/II; late: stage III/IV.

**Figure 2.**
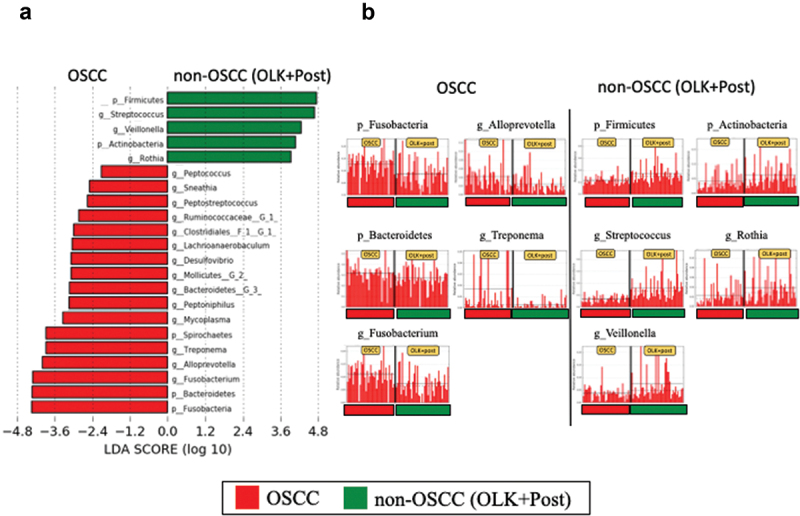
The results of LEfSe analysis comparing between oral squamous cell carcinoma (OSCC) and non-OSCC groups (OLK, oral leukoplakia OLK: Post, post-operative of OSCC). (a) LEfSe comparison between OSCC and non-OSCC groups. (b) Top 5 bacteria of high linear discriminant analysis (LDA) for each group.

LEfSe comparing between OSCC and non-OSCC (OLK+Post) groups showed that high LDA scores were observed for the phylum *Fusobacteria*, genus *Fusobacterium* and phylum *Bacteroidetes* in the OSCC group and for the phylum *Firmicutes*, and genus *Streptococcus* in the non-OSCC groups (Figure 2(a,b)). The summary of LEfSe results is shown in Table 4. In OSCC group, high LDA score was identified for the genus *Streptococcus* in the OSCC early group and for the genus *Fusobacterium* in the OSCC late group. These results revealed that, compared with the other groups, the relative abundance of the phylum *Fusobacteria*, genus *Fusobacterium* and phylum *Bacteroidetes* significantly increased in the OSCC group and that of the phylum *Firmicutes* and genus *Streptococcus* significantly decreased. Therefore, these bacteria may be cancer-specific, highlighting as potential agent candidate for OSCC detection.

**Table 4.** Summary of LEfSe analysis.

	Up (top 3 of LDA score >4.0)			Down (top 3 of LDA score >4.0)		
Comparison group	1	2	3	1	2	3
OSCC						
vs OLK/ Post*	p *Fusobacteria*	p *Bacteroidetes*	g *Fusobacterium*	p *Firmicutes*	g *Streptococcus*	g *Veillonella*
vs OLK	p *Fusobacteria*	g *Fusobacterium*	g *Treponema*	p *Firmicutes*	g *Streptococcus*	g *Mesorhizobium*
vs Post	p *Bacteroidetes*	g *Fusobacterium*	p *Fusobacteria*	p *Firmicutes*	g *Streptococcus*	g *Veillonella*
OSCC late						
vs OSCC early	g *Fusobacterium*	g *Alloprevotella*	g *Catonella*	g *Streptococcus*	none	none
Rec						
vs non-Rec	p *Fusobacteria*	g *Fusobacterium*	none	g *Streptococcus*	none	none

Abbreviations: LefSe: linear discriminant analysis of effective size; LDA: linear discriminant analysis; OSCC: oral squamous cell carcinoma; OLK: oral leukoplakia; Post: post operative of OSCC; p: phylum; g: genus; late: stage I/II; early: stage III/IV; Rec: recurrence.*This analysis means that significantly increased abundance of top 3 bacteria (left column) and decreased abundance of bacteria (right column) in OSCC group when compared to OLK and Post group.

**Figure 3.**
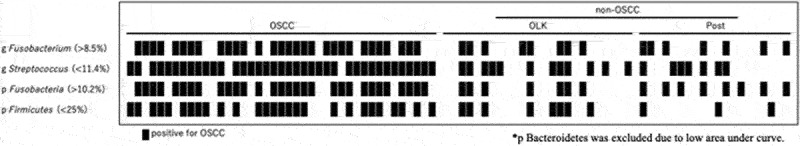
A panel of bacterial markers for OSCC and non-OSCC including more than two candidate bacteria showing the different distribution of OSCC positive bacterium between each group.

**Figure 4.**
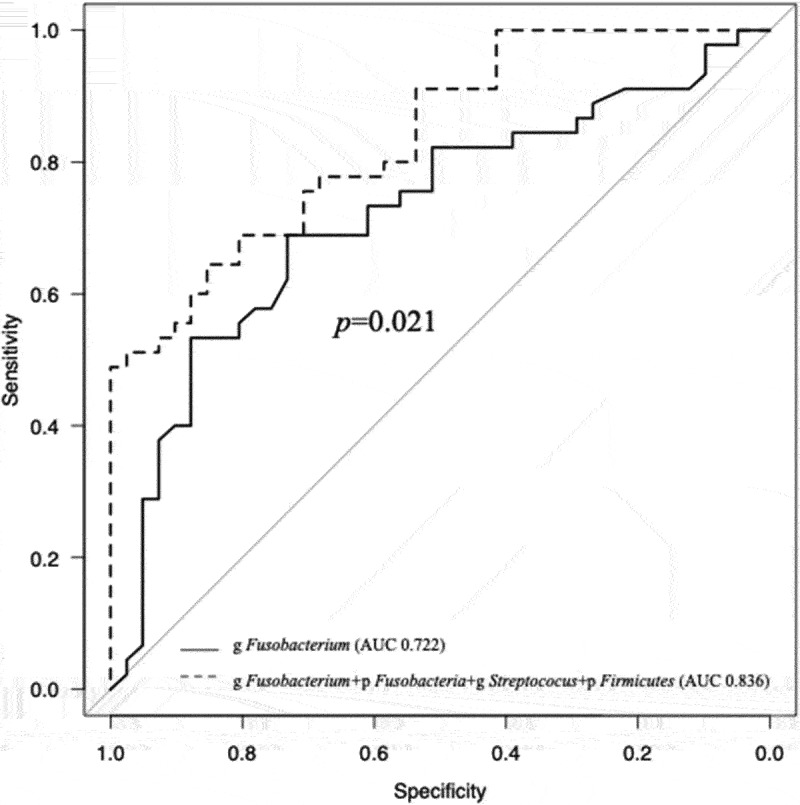
The result of multivariate ROC curve analysis showing a significant high AUC value in combination of four candidate bacteria when compared with g *Fusobacterium* alone.

**Figure 5.**
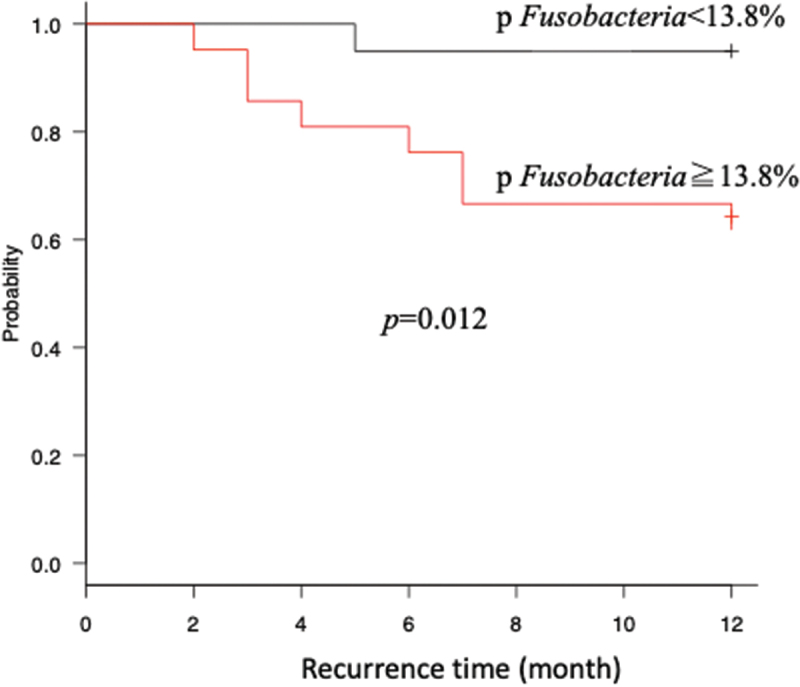
Kaplan-Meier curve showing recurrence within 1 year in oral squamous cell patients with high relative abundance of p *Fusobacteria* versus low abundance of it.

For candidate bacteria, the optimal cut-off values of bacterial relative abundance to discriminate the OSCC and non-OSCC groups based on ROC curve analysis are as follows: 10.2%, 8.5%, 25.8%, 11.4% and 25% for the phylum *Fusobacteria*, genus *Fusobacterium*, phylum *Bacteroidetes*, genus *Streptococcus* and phylum *Firmicutes*¸ respectively (Table 5). Phylum *Bacteroidetes* was excluded for further investigation due to low area under the curve (AUC) value. The sensitivity and specificity of using one candidate bacterium were not enough good. To improve the sensitivity and specificity for OSCC distinction, we have made a panel of bacterial markers for OSCC and non-OSCC including more than two candidate bacteria (Figure 3). Using more than two bacteria revealed most high sensitivity for OSCC detection (Table 6). Moreover, AUC was significantly high when combined four candidate bacteria compared with g *Fusobacterium* alone (Figure 4). We treated OLK and post groups as non-OSCC group, because there were few differences of diversity and LEfSe analyses between OLK and Post groups (data not shown). In univariate analysis of the OSCC and non-OSCC groups, significant differences were observed in bacterial relative abundances by the cut-off value. Moreover, in multivariate analysis adjusted by age, sex, smoking and drinking status and remaining number of teeth, the presence of OSCC demonstrated highly correlation with the fluctuation of each candidate bacteria (Table 7). In the patients who exhibited recurrence or cervical metastasis within 1 year postoperatively (recurrence group), LEfSe revealed that high LDA score was observed for the phylum *Fusobacteria*, genus *Fusobacterium* in the recurrence group and for the genus *Streptococcus* in the non-recurrence group. Kaplan-Meier curve revealed that high relative abundance of phylum *Fusobacteria* group (above the median: 13.8%) had significantly recurred within 1 year postoperatively (Figure 5).

**Table 5.** The results of ROC curve analysis between OSCC and non-OSCC.

Candidate bacteria for OSCC	cut off value (%)	sensitivity/specificity	AUC		
p *Fusobacteria*	≧10.2	0.689/0.732	0.703		
g *Fusobacterium*	≧8.5	0.689/0.732	0.722		
p *Bacteroidetes*	≧25.8	0.829/0.533	0.632		
p Firmicutes	<25.0	0.756/0.659	0.745		
g *Streptococcus*	<11.4	0.578/0.928	0.831		

Abbreviations: ROC: receiver operating characteristic; OSCC: oral squamous cell carcinoma; AUC: area under curve.

**Table 6.** The sensitivity and specificity for OSCC detection when combined more than two bacteria.

Number of positive bacterium for OSCC	sensitivity/specificity
more than two bacteria	0.902/0.622
more than three bacteria	0.634/0.800
four bacteria	0.512/0.844

Abbreviations: OSCC: oral squamous cell carcinoma.*p Bacteroidetes was excluded due to low area under curve value.

**Table 7.** Univariate and multivariate analysis of candidate OSCC-associated bacteria between OSCC and non-OSCC groups.

	Univariate analysis			Multivariate analysis*	
Factor	OSCC	Non-OSCC (OLK+Post)	*P-value*	OR (95%CI)	*P-value*
	(n = 41)	(n = 45)			
Abundance of p *Fusobacteria*					
≧10.2%	30	15	0.0003	5.04 (1.80–14.20)	0.002
<10.2%	11	30		1[reference]	
Abundance of g *Fusobacterium*					
≧8.5%	30	15	0.0003	5.64 (2.00–015.90)	0.001
<8.5%	11	30		1[reference]	
Abundance of p *Firmicutes*					
<25%	27	11	0.0002	6.88 (2.40–19.70)	<0.001
≧25%	14	34		1[reference]	
Abundance of g *Streptococcus*					
<11.4%	38	19	<0.0001	24.5 (5.65–107.0)	<0.001
≧11.4%	3	26		1[reference]	

Abbreviations: OSCC: oral squamous cell carcinoma; OLK: oral leukoplakia; Post: post operative of OSCC; OR: odd’s ratio; CI: confidence interval; p: phylum; g: genus.

*adjusted by age, sex, smoking status, drinking status, and number of teeth.

## Discussion

In the present study, analyses of saliva samples revealed that oral microbiome profiles significantly differed between OSCC, OLK and post groups. The abundances of bacteria characteristic to OSCC were identified to serve a possibility of novel candidate biomarker for OSCC detection. Till date, only few studies have assessed microbiome profiles to identify diagnostic tools. The occurrence of 15% of OSCC cannot be explained by the major risk factors, such as smoking, alcohol consumption and virus infection [6]. In such patients, it has been suggested that poor oral hygiene induces OSCC development; however, there is a lack of evidence. Increase of unfavorable bacteria for healthy oral environment may play an important role in OSCC development. We hypothesized that oral microbiome specific to OSCC can be used as novel OSCC detection tools. Indeed, recent studies have shown that microbiome shifts in the oral cavity, stomach, and gut are highly associated with the initiation and progression of various malignant tumors [7–9]. Oral commensal bacteria were detected in metastatic cervical lymph node in patients with OSCC, suggesting that oral bacteria may associate with OSCC progression [10]. NGS methods have allowed analysis of bacterial flora (including anaerobic bacteria) and have revealed that several bacteria cooperatively act in the development and progression of diseases [11,12]. Therefore, regarding investigation of the association between OSCC and oral bacteria, a comprehensive analysis of microbiome profiles is necessary. Saliva samples are suitable for analysis as they may reflect the microbiome profile of the whole oral cavity and multiple exposure factors [13–15].

In this study, significant differences were observed in the abundances of the genus *Streptococcus, Aggregatibacter* and *Alloprevotella* among the three groups. *Streptococcus* was the second most dominant genera (11.8%) and should therefore be regarded as important. In this study, the relative abundance of phylum *Firmicutes* was low as compared with other studies. This may cause the differences of saliva sampling time, collection kit, using Database or amplification 16S rRNA region. For diversity analysis of saliva samples, the results revealed that the bacterial flora differed among groups. This result may indicate that the presence of diseases, such as OSCC and OLK, and post-operative factors, influence the diversity of bacterial flora, leading to unique microbiome profiles. In this study, it was unclear that whether these differences in bacterial flora were responsive or causative for disease. However, Pushalker et al. [16] reported that the continuous presence of bacteria at tumor samples in the oral cavity suggested a role of these bacteria in OSCC progression or invasion. Our results revealed that differences of bacterial diversity between early and advanced stages from OSCC saliva samples. This may be due to the effect triggered by tumor progression, favoring the growth conditions such as tissue necrosis of a subset of microbes.

We demonstrated a significant difference in the abundances of the bacterium between the OSCC and non-OSCC groups using LEfSe analysis. High LDA scores reflect significantly higher abundance of certain taxa. Therefore, these bacteria were selected as the candidates for novel OSCC detection tools. Although ROC curves were used to determine optimal cut-off values to distinguish among groups based on candidate bacterial abundances, there remain no criteria for the same. Moreover, a significant shift in the bacterial abundance of the genus *Fusobacterium* and *Streptococcus* from patients with advanced OSCC were shown; these bacteria may have an impact on OSCC development.

In univariate analysis of each candidate bacterium, significant differences were found in each bacterial cut-off values when compared the OSCC and non-OSCC groups. Because periodontal status may affect bacterial flora composition as a contributing factor, we included the remaining number of teeth for predicting periodontal status. Takeshita et al. [17] reported that significant differences of oral microbiome were observed based on the remaining number of teeth (above or below 9). In our study, most patients possessed 9 or more teeth, and there was no significance in the number of remaining teeth among the groups (data not shown). Therefore, periodontal status appeared to be consistent, and we considered it to have no influence on comparison of bacterial flora. Furthermore, the results of multivariate analysis revealed that the presence of OSCC had strong impact for fluctuation of each candidate bacterium. This suggested that the difference of relative abundances of each candidate bacterium can detect the existence of OSCC and that the oral microbiome profile can be a useful OSCC detection tool. In addition, examination in patients who had early recurrence demonstrated that candidate bacteria had significance in recurrent (+) group, supporting their use as prognostic factors. We revealed that high relative abundance of phylum *Fusobacteria* group had significantly recurred within 1 year postoperatively. This result may be influenced by the association in carcinogenesis of phylum *Fusobacteria*.

The mechanisms of oral microbiome association in carcinogenesis may include the induction of chronic inflammation that causes immunosuppression, direct or indirect interference with eukaryotic cell cycle and signal pathways, the production of carcinogens such as acetaldehyde, the inhibition of cellular apoptosis, and the activation of cell proliferation and the promotion of cellular infiltration [9,18]. Of these, persistent chronic inflammation is considered to play a pivotal role in all stages of carcinogenesis as inflammatory cells and cytokines elicited by chronic inflammation produce reactive oxygen and nitrogen species, which can cause DNA mutation [19,20]. Phylum *Fusobacterium* associates with the induction of chronic inflammation, and promotion of cellular invasion, which may induce high recurrence rate. Furthermore, the production of inflammatory cytokines in immunodeficiency status can cause to further promote cell proliferation and infiltration, leading to the inhibition of tumor cell suppression [21,22].

Saliva collection is non-invasive and easy; thus, it is suitable for screening of diseases, including OSCC. The oral bacterial flora in saliva has been reported to be both stable and highly reproducible and is not greatly affected by recent dental treatment, tooth brushing or food intake [23,24]. Saliva is a final product of blood, and various tumor markers can be detected in saliva at the same levels similar to those in blood [25,26]. Thus, it may be possible to detect systemic changes from saliva. Hu et al. [27] reported that a decrease in the abundance of the phylum *Proteobacteria* may be used as a novel diagnostic marker of gastric cancer. The oral bacterial flora in saliva displays a circadian rhythm, which varies depending on specific bacterial species [28–30]. Therefore, if the saliva sample collection time differs, the bacterial species and abundance may fluctuate. In the present study, all saliva samples were collected under same conditions, thus potentially minimizing biases of bacterial circadian rhythm.

In conclusion, this study demonstrated that the differences of microbiome profiles from saliva such that the phylum *Fusobacteria*, genus *Fusobacterium* and phylum *Bacteroidetes* significantly increased and the phylum *Firmicutes* and genus *Streptococcus* significantly decreased in the OSCC group compared with non-OSCC groups; these may have potential for novel OSCC detection tools. In addition, our examination of patients who had early recurrence suggested that the potential of oral microbiome as a prognostic factor. Our findings may facilitate the possibility of a clinically applicable novel biomarker that can contribute to early detection of OSCC and for prediction of malignant change from oral potentially malignant disorders. This study cannot clarify how the candidate bacterial biomarkers for OSCC affect carcinogenesis and the progression of tumor, but the candidates may have potential roles for extraction of high-risk case of oral leukoplakia or recurrence of postoperative patients for OSCC. Further prospective study will be warranted for ensuring the value of oral microbiome profiling among oral diseases.
